# Sex Hormones Influence the Helmholtz–Kohlrausch Effect

**DOI:** 10.18502/jovr.v19i1.15441

**Published:** 2024-03-14

**Authors:** Brian K. Foutch

**Affiliations:** ^1^University of the Incarnate Word, Rosenberg School of Optometry, San Antonio, TX, USA

**Keywords:** Brightness, Contraception, Helmholtz–Kohlrausch Effect, Hormones, Luminance, Menstrual Cycle, Saturation

## Abstract

**Purpose:**

Saturated lights appear brighter than white lights of the same luminance. This is the Helmholtz–Kohlrausch (H–K) effect, and the phenomenon can be estimated by modeling achromatic luminance and saturation to total brightness. Current H–K effect models are different between women and men and are also more variable in women, which may be due to hormonal changes across the menstrual cycle (MC).

**Methods:**

Total brightness (B) and achromatic luminance (L) were measured across blue, green, yellow-green, yellow, and red hues. These data were measured along with salivary hormone levels for nine cycling women and seven oral contraceptive (OC) users at points representing the menstrual, peri-ovulation, and luteal phases.

**Results:**

Simple brightness/luminance (B/L) ratio estimates of the H–K effect did not differ by OC use or MC phase, but B/L ratios were higher for the red stimulus in cycling women than OC users during the luteal phase. Estrogen, progesterone, and their interaction predicted 18% of the variation in brightness for cycling women. For OC users, only estrogen could be fit to brightness models where it accounted for 5% of brightness variance.

**Conclusion:**

These findings first provide clear support for separating cycling women from OC users, particularly when examining long-wavelength mechanisms. Next, the interaction of OC use and MC phase on B/L ratios for the red stimulus adds to a rich history of long-wavelength mechanisms. Lastly, the current result amends previous brightness models with multiple hormone terms for cycling women but not OC users.

##  INTRODUCTION

There is robust evidence that female visual systems are better tuned than males for chromatic stimuli.^[1,2,3,4,5,6,7,8,9]^ Some of these differences are organizational, so it would be a gross error to fully attribute sex differences to sex hormones or the menstrual cycle. ${\;}^{\left[10,11\right]}$ However, equivocal reports of menstrual cycle effects on color perception are interesting. Finkelstein and Lorenzetti found restricted chromatic visual fields and decreased green and yellow sensitivity during the menstrual phase.^[12,13]^ Other studies have demonstrated stable achromatic visual fields across the menstrual cycle,^[14,15,16]^ but have suggested decreased sensitivity to blue stimuli during the high hormone luteal phase.^[14,15]^ Observations of cyclical effects on wavelength-specific photoreceptor mechanisms have revealed cyclical effects for blue but not green or red mechanisms,^[17]^ while color discrimination has been found to be best near ovulation.^[18]^

**Figure 1:**
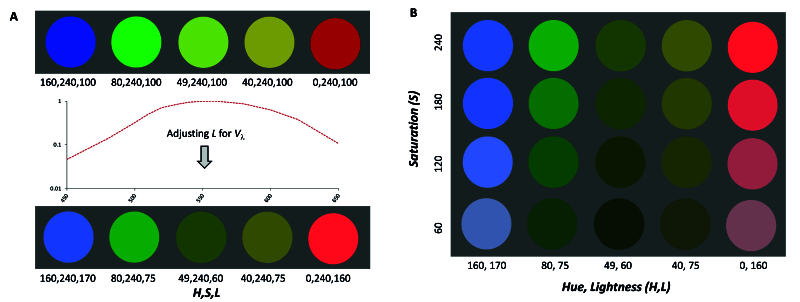
The Helmholtz–Kohlrausch effect explained. The colored patches in both (A) and (B) loosely represent the blue (450 nm), green (520 nm), yellow-green (560 nm), yellow (580 nm), and red (650 nm) stimuli used in the current experiment. In the top row of (A), the stimuli all have a fixed saturation (S = 240) and lightness (L = 100) in HSL coordinates. Since luminance is most efficiently processed by the standard observer for yellow-green but least for the blue and red stimuli, lightness is adjusted (i.e., increased for red and blue stimuli and decreased for the remaining stimuli) in the bottom row of (A) according to the standard luminous efficiency curve (V-lambda). For most observers, the static brightness in the bottom row of (A) will then appear to be greatest for blue and red stimuli and least for the yellow-green or yellow stimuli. The top row of (B) is the same as the L-adjusted (bottom) row of (A). When the saturation (S) is decreased in (B) the apparent brightness decreases for all stimuli.

The perception of colored objects is complex, occurring via distinct visual pathways.^[19]^ Fortunately, these contributions can be quantified by methods such as heterochromatic flicker (HFM) and direct brightness (DBM) matches. In HFM, observers minimize flicker between a color and reference stimulus. If the flicker rate is fast enough, slower chromatic pathways are avoided, and equally luminous stimuli will appear to be continuous. HFM are additive (i.e., luminance of a mixture of colors A and B should be equal to the sum of luminance A and luminance B) and used to form luminous efficiency functions used in calibrating displays.^[20]^ During DBM, observers adjust the intensity of the color field until it matches in brightness to the reference field. Direct brightness matches are not additive and are more difficult than HFM for most observers.^[21]^ Additivity failures from the chromatic channels enhance brightness, especially for saturated short (blue) or long (red) wavelengths.^[22]^ Brightness is less enhanced in less saturated (i.e., whiter) colors or combinations of colors such as yellow. This complex effect of saturation on perceived brightness is known as the Helmholtz–Kohlrausch (H–K) effect [Figure 1]. ^[23,24]^**

The H–K effect is not perfectly understood, but—at a minimum—it can be derived from the ratio of chromatic to achromatic activation.^[25]^ To better estimate the effect, however, one needs to consider a non-linear brightness-luminance relationship such as: *log B = a ${\;}_{0}$ + a ${\;}_{1}$ logL + a ${\;}_{2}$ S ${\;}_{\lambda }$ *, where *B* = brightness, *L* = luminance, *S ${\;}_{\lambda }$* = saturation, *a ${\;}_{0}$* = constant, and *a ${\;}_{1}$ , a ${\;}_{2}$* are the coefficients for achromatic luminance and saturation, respectively. A recent sex comparison of the H–K effect revealed: a clear female advantage in the effect, a larger saturation coefficient for women, that saturation predicted more variance in perceived brightness in women, and that the overall model better predicted brightness for men.^[3]^ Decreased fit of the model for women suggests a within-females variation that may be associated with cyclical or hormonal changes. After all, estrogen has significant effects on human physiology outside of reproduction,^[26]^ and estrogen receptor proteins have been observed in human ocular structures.^[27,28]^ Progesterone receptors have also been localized in mammalian retinas.^[29]^

Endogenous sex hormone levels vary predictably across the menstrual cycle,^[30]^ but can be exogenously modulated with selective estrogen receptor modulators (used as breast cancer therapy) or more commonly with oral contraceptives (OC), both of which have also been associated with color perception changes.^[31,32,33,34]^

Idealized menstrual cycles last approximately 28 days, are accompanied by large perturbations in the primary form of estrogen (17 β -estradiol; estradiol or E2) and progesterone (P) levels, and are divided into follicular (days 1–14; low E2, low P) and luteal (days 15–28; high E2, high P) phases.^[35]^ The follicular phase can be further divided into the menstrual (days 1–7 of the cycle; low E2, low P) and late follicular (days 7–14; increased E2 but low P) phases. At the cycle midpoint is the peri-ovulatory phase ( ∼ day 13; preceded by a first E2 peak but low P). Understanding this pattern allows researchers to assume categorical E2 and P levels when examining their effects on perception or behavior,^[30,36]^ which is the purpose of the current investigation; to determine whether the H–K effect is higher in cycling women and during phases of the menstrual cycle when hormone levels are higher.

##  METHODS

### Subjects

Eligible women had normal visual acuity ( ≥ 20/20), normal color vision using pseudo-isochromatic plates and Medmont C-100, and were not pregnant. Hormonal contraceptive use was permissible. The institutional review board of the University of Missouri, St. Louis approved the protocol (HSC Approval #: 0600506F), and the investigation was carried out following the tenants outlined in the 2013 Amendments to the Declaration of Helsinki. Informed consent was obtained from 16 women (ages 21–40 years; nine experiencing normal cycles [ages 25.8 ± 3.2 years] and seven using combination OC [ages 26.0 ± 6.2 years]).

### Sessions

Sessions were scheduled during the menstrual (days 1–7), peri-ovulation ( ∼ day 12), and luteal ( ∼ day 21) phases. Follicular phases vary a great deal between women, but the typical luteal phase is 14 days^[37]^ with peak estrogen levels occurring 16 days before the start of the next cycle.^[35]^ Peri-ovulatory and luteal sessions were then scheduled 16 and 7 days, respectively, before the predicted start of the next cycle. Experimental sessions not completed within three days of the prediction were rescheduled during the next month. OC users do not experience hormonal cycles, but data were collected at similar times. Three cycling women and one OC user did not complete sessions during peri-ovulation.

### Hormone Measures

Participants collected saliva samples (ZRT Laboratory, Beaverton, OR) at home on the day of each experimental session prior to eating, drinking, or brushing their teeth. The collected samples were brought to each session and mailed that day. Progesterone was measured with a direct competitive radioimmunoassay (RIA) and estradiol via by double antibody RIA.

### Apparatus

An open-view system was used to produce a 2.5º circular field. A uniform field was used for HFM; a side-by-side bipartite field was used for DBM. For the HFM task, the test and reference beams were temporally separated via a mirror rotating at 18 cycles/sec (Hz) and illuminated an acrylic cylinder which served as a 1.9 cm diffuse circular viewing screen. For the DBM task, the reference beam was reflected onto the left viewing half of a bipartite viewing field, separated from the color test field by a 0.5 mm aluminum sheet.

### Procedure

After adapting to low background luminance for 5 mins, participants practiced matches, concluding when trials at each test wavelength—450, 520, 560, 580 and 650 nm—fell within one standard deviation of the mean. Participants adapted to the 5 cd/m^2^ reference field for 30 sec prior to testing at each color then adjusted the color stimulus intensity until it matched the reference field (for DBM) or minimized the flicker sensation (for HFM).

### Data Analysis

Relative luminosity (RL) was calculated at each wavelength by dividing the reference luminance by the average luminance of four trials. DBM values were used as estimates of total brightness (i.e., chromatic + achromatic activation), indicated further here by the letter B. HFM values were used as estimates of perceived luminance (i.e., achromatic activation), represented here by the letter L. For regression models, saturation was calculated as S ${\;}_{\lambda }$ = 13([*u ${\;}_{n}$ –u' ${\;}_{n}$ *]^2^ – [*v ${\;}_{n}$ –v' ${\;}_{n}$ *]^2^) ${\;}^{1/2}$ , where (*u ${\;}_{n}$ ,v ${\;}_{n}$ *) and (*u' ${\;}_{n}$ ,v' ${\;}_{n}$ *) are the chromaticity values of the color and reference broadband stimuli, respectively, in CIELUV space. The normalized saturation values were: 1.00 (blue at 450 nm), 0.60 (green at 520 nm), 0.31 (yellow-green at 560 nm), 0.29 (yellow at 580 nm), and 0.99 (red at 650 nm).

**Figure 2:**
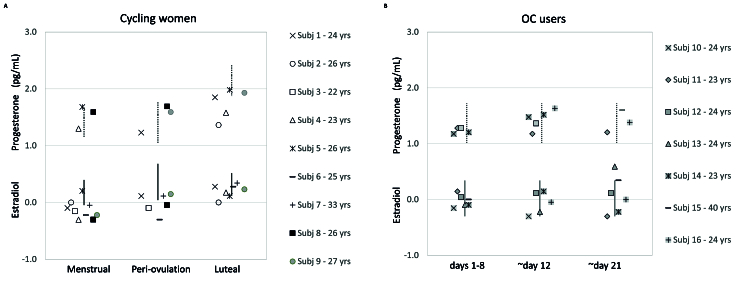
Logarithmic salivary estradiol and progesterone levels across the menstrual cycle for cycling women (A) and oral contraceptive users (B). The vertical bars represent reference hormone ranges for estradiol (solid) and progesterone (dotted).

Plots of salivary estradiol and progesterone across the menstrual phase were visually compared with established laboratory reference values [Figure 2]. Reduced number of progesterone assays constrained the analysis to univariate analysis of variance (ANOVA) with OC use and menstrual cycle (MC) phase as fixed factors. The within subjects effects of MC phase and between subjects effects of OC use on estradiol levels and B/L ratios were examined via repeated measures (RM) ANOVA. Post-hoc comparisons were used to determine pairwise differences in hormone levels and B/L ratios between menstrual, peri-ovulation, and luteal phases as well as between cycling women and OC users for all five hues across the three menstrual cycle phases.

Lastly, estradiol and progesterone terms were added to the saturation regression model for all participants and for cycling women and OC users separately. All statistical analyses were performed using SPSS for Windows (SPSS Inc., Chicago, IL).

##  RESULTS

### Salivary Estradiol and Progesterone Levels

Data were collected on the following days for cycling women: menstrual (3.7 ± 2.0), peri-ovulation (11.5 ± 0.8), and luteal (19.7 ± 1.6) and for OC users: menstrual (4.0 ± 2.8), peri-ovulation (11.8 ± 1.8), and luteal (19.4 ± 1.5). Kolmogorov–Smirnov tests revealed that estradiol (*P* = 0.009) and progesterone measures (*P* = 0.046) were not normally distributed. Log-transformed estradiol (*P* = 0.200) and progesterone (*P* = 0.564) levels were used for all analyses. All other measures were normally distributed.

Estradiol levels were lower than reference ranges (*www.zrtlab.com/resources/reference-documents/saliva-reference-ranges*) for cycling women during the menstrual phase and peri-ovulation [Figure 2]. Progesterone levels were lower than reference ranges for cycling women during the luteal phase. Hormone levels were within reference ranges for OC users.

Estradiol levels were equivalent between cycling women and OC users (F [1,7] = 0.49, *P* = 0.507). Estradiol levels varied significantly across MC phases for all participants (*F* [2,14] = 5.68, *P* = 0.016, η ^2^ = 0.45) and were higher on pairwise comparisons during the luteal phase than the menstrual phase for all women (t ^[16]^ = 3.88, *P* = 0.003). For cycling women, estradiol levels were also different across MC phases (*F* [2,8] = 13.01, *P* = 0.003, η ^2^ = 0.77), where luteal levels were higher than menstrual phase levels (t ^[8]^ = 7.10, *P* = 0.0001). Estradiol levels were equivalent between MC phases for OC users (*F* [12,6] = 0.713, *P* = 0.519).

Progesterone levels were higher for cycling women (*F* [1,18] = 10.31, *P* = 0.001, η ^2^ = 0.19) but were the same across phases for all participants (*F* [2,18] = 2.06, *P* = 0.157), cycling women (*F* [2,12] = 1.81, *P* = 0.214), and OC users (*F* [2,9] = 2.00, *P* = 0.191). Luteal levels were higher than during the menstrual phase (t ^[13]^ = 2.18, *P* = 0.048) for all participants, however, there were no pairwise progesterone differences between MC phases.

### Effects of Contraception and Menstrual Cycle Phase on B/L Ratios

**Figure 3:**
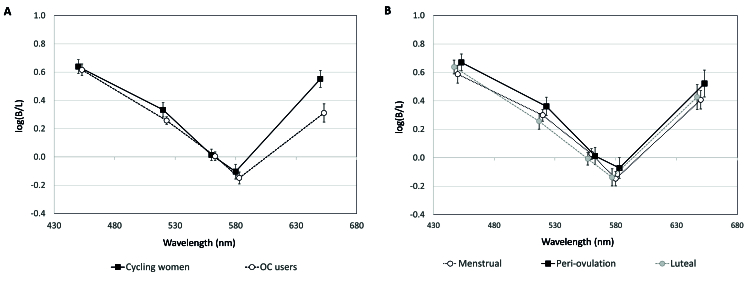
Mean B/L (brightness/luminance) ratios for all participants by OC use (A) and menstrual cycle phase (B). Error bars = ± 1 SEM.

B/L ratios were slightly higher for cycling women than OC users, however, the difference did not reach significance (*F* [1,10] = 1.83, *P* = 0.206) [Figure 3A].

**Figure 4:**
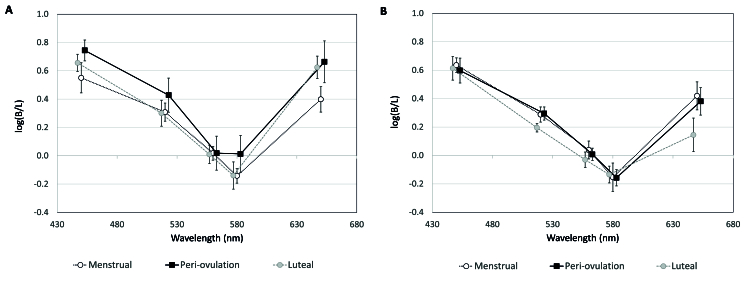
Mean B/L (brightness/luminance) ratios for cycling women (A) and OC users (B). Error bars = ± 1 SEM.

B/L ratios appeared higher at 650 nm for cycling women than OC users [Figure 3A]. This was confirmed on pairwise comparison (t ^[43]^ = 2.73, *P* = 0.009). There were no main within-subjects effects of MC phase (*F* [2,20] = 2.01, *P* = 0.160) [Figure 3B]. There were also no pairwise differences between phases [Figure 3B]. B/L ratios for cycling women and OC users are shown separately in Figure 4. There were no main effects of MC phase for cycling women (*F* [2,10] = 1.18, *P* = 0.347) [Figure 4A] nor OC users (F [2,10] = 2.00, *P* = 0.186) [Figure 4B]. There were no pairwise differences between phases in cycling women.

For OC users, B/L ratios were reduced ∼ day 21 compared to other “phases” [Figure 4B], however, there was only a strong trend toward lower B/L ratios compared to days 1–8 at 650 nm (t ^[10]^ = 2.05, *P* = 0.068). Lastly, there was an interaction effect of OC use and MC phase at 650 nm, where B/L ratios were higher for cycling women during the luteal phase than for OC users around day 21 (t ^[10]^ = 2.70, *P* = 0.022).

### Regression Models of Estradiol and Progesterone on B/L Ratios

Linear regressions were calculated to predict total brightness (B) based on luminance (L), saturation (S ${\;}_{\lambda }$ ), estradiol (E2), and progesterone (P). The regressions were performed stepwise to determine the effects of first luminance, then saturation, then lastly hormone levels. This was accomplished for all participants, cycling women, and OC users (summarized in Table 1). For all participants, the luminance only model (i.e., *B = a ${\;}_{0}$ + a ${\;}_{1}$ logL*) was significant but only predicted 29% of the variance in brightness. Adding saturation (S ${\;}_{\lambda }$ ) to the regression model resulted in 22% of additional predicted variance (i.e., ∇ R^2^). Adding the hormone terms (logE2, logP, logE2 × logP) resulted in an ∇ R^2^ of 0.08 and an overall significant regression equation (*F* [5,114] = 36.5, *P* = 0.001, R^2^ = 0.59).

**Table 1:** Regression results for all participants, cycling women, and OC users using brightness (B) as the criterion.


**Predictors**	Coefficient*	**SE**	**95% CI**		**Sig (** ***P*** **-value)**	**Model fit (R^2^)**	** ∇ R^2^**
			**LL**	**UL**			
*All participants*							
Constant (a ${\;}_{0}$ )	–0.76	0.14	–1.04	–0.47	0.001	–	–
Luminance (logL)	1.07	0.10	0.89	1.26	0.001	0.29 †	–
Saturation (S ${\;}_{\lambda }$ )	1.04	0.12	0.79	1.28	0.001	0.51 ‡	0.22
Estradiol (logE2)	3.50	0.75	2.02	4.97	0.001	–	–
Progesterone (logP)	0.28	0.10	0.09	0.47	0.004	–	–
Interaction (logE2 × logP)	–2.40	0.50	–3.38	–1.42	0.001	0.59 ${\;}^{§}$	0.08
*Cycling women*							
Constant (a ${\;}_{0}$ )	–1.13	0.20	–1.52	–0.73	0.001	–	–
Luminance (logL)	1.07	0.13	0.82	1.31	0.001	0.18 †	–
Saturation (S ${\;}_{\lambda }$ )	1.13	0.16	0.82	1.44	0.001	0.55 ‡	0.37
Estradiol (logE2)	5.19	1.04	3.16	7.23	0.001	–	–
Progesterone (logP)	0.52	0.13	0.26	0.77	0.001	–	–
Interaction (logE2 × logP)	–3.66	0.68	–4.98	–2.34	0.001	0.73 ${\;}^{§}$	0.18
*OC users*							
Constant (a ${\;}_{0}$ )	–0.28	0.06	–0.40	–0.15	0.001	–	–
Luminance (logL)	0.90	0.13	0.64	1.16	0.001	0.48 †	–
Saturation (S ${\;}_{\lambda }$ )	0.69	0.19	0.32	1.05	0.001	0.59 ‡	0.11
Estradiol (logE2)	0.34	0.14	0.08	0.61	0.014	–	–
Progesterone (logP)	–	–	–	–	*ns*	–	–
Interaction (logE2 × logP)	–	–	–	–	*ns*	0.64 ${\;}^{§}$	0.05
	
	
CI, confidence interval; E2, estradiol; LL, lower limit; *ns,* not significant; P, progesterone; SE, standard error; Sig, statistical significance; UL, upper limit *Coefficients for final models containing all terms; Model Predictors: † a ${\;}_{0}$ + a ${\;}_{1}$ log(L); ‡ a ${\;}_{0}$ + a ${\;}_{1}$ log(L) + a2s ${\;}_{\lambda }$ ; ${\;}^{§}$ a ${\;}_{0}$ + a ${\;}_{1}$ log(L) + a ${\;}_{2}$ s ${\;}_{\lambda }$ + a ${\;}_{3}$ logE2 + a ${\;}_{4}$ logP + a ${\;}_{5}$ logE2 x logP							

For cycling women, all models were also significant [Table 1]. The luminance only model only predicted 18% of the variance in B and adding saturation to the regression model resulted in a total R^2^ of 0.55 ( ∇ R^2^ of 0.37). Adding the hormone terms resulted in an ∇ R^2^ of 0.18 and an overall significant regression equation (*F* [5,59] = 27.0, *P* = 0.001, R^2^ = 0.73).

**Figure 5:**
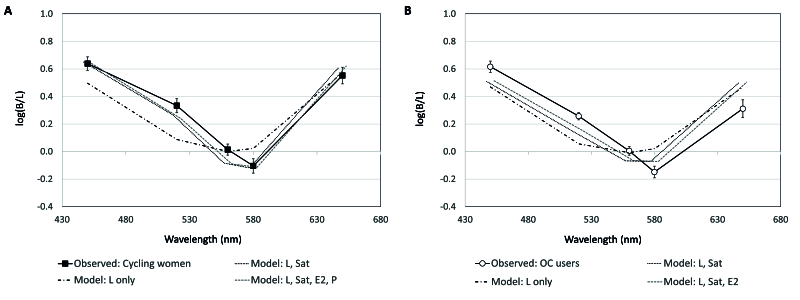
Observed and modeled B/L (brightness/luminance) ratios for cycling women (A) and OC users (B). Error bars = ± 1 SEM.

For OC users, the luminance only model was significant and predicted a much higher portion (48%) of the variance in brightness than for cycling women. Adding saturation to the regression model only resulted in a total R ${\;}^{2}$ of 59% ( ∇ R^2^ = 0.11). When adding E2, *P*, and the interaction term stepwise individually, a significant regression equation was found for luminance, saturation, and E2 (*F* [3,51] = 26.0, *P* = 0.001, R^2^ = 0.64; i.e., adding E2 to the regression model only resulted in a ∇ R^2^ of 5%). Modeled B/L ratios for cycling women and OC users are plotted along with observed values in Figure 5.

##  DISCUSSION

### Notable Results

The most notable finding is that brightness models differed between cycling women and OC users [Figure 5]. In cycling women, luminance, and hormone terms each predicted 18% while saturation predicted 37% of the variance in brightness. This contrasts with OC users, where luminance predicted 48% and saturation only predicted 11% of the variance in brightness. Estradiol only predicted ∼ 5% of brightness in OC users, and progesterone could not be successfully modeled to brightness. B/L ratios for cycling women from models including saturation and/or luminance slightly underestimated observed ratios [Figure 5A], however, they more closely aligned to observed ratios than data modeled for OC users [Figure 5B]. The only stimulus modeled better for OC users was yellow-green (560 nm). This is not surprising. Models in OC users rely more heavily on luminance, and the peak luminous efficiency for most observers is near 560 nm (i.e., yellow-green).^[20]^

The second notable finding can be seen in Figure 3A, where B/L ratios were significantly higher at 650 nm. This finding (*P* = 0.009) for the red stimulus survives a conservative Bonferroni post-hoc correction for five comparisons (adjusted *P* = 0.045) and represents a large effect (Cohen's d = 0.82). There were no such B/L differences for the red stimulus in a previous study by the present author.^[3]^ However, that study compared women at random days. In the present study, data for cycling women were systematically collected from all phases and compared with similar data from OC users. When doing so, the lowest B/L ratios at 650 nm for cycling women were during the menstrual phase [Figure 4A]. This implicates estradiol, which was lowest during the menstrual phase for cycling women [Figure 2A]. B/L ratios were lowest for OC users during the luteal phase [Figure 4B] when estradiol and progesterone levels were lower than in cycling women [Figure 2B]. The clear interaction of luteal phase and OC use further implicates progesterone in the difference for the red stimulus.

While exploratory, the present B/L ratio differences between cycling women and OC users for the red stimulus during the luteal phase may be explained by physiological effects. For example, ovulation usually does not occur in OC users, and the corpus luteum decays and stops secreting progesterone.^[36]^ The synergism of elevated E2 and P levels during the luteal phase causes a slight increase in basal body temperature during the luteal phase.^[38]^ de Vries and St. George demonstrated that an increase in body temperature increased threshold sensitivity to red light. ${\;}^{\left[39,40\right]}$ Temperature was not measured in the present study, so any effect of body temperature remains a speculation. Increased ionic absorption increases cone photoreceptor excitation and leads to increased neurotransmitter release and enhanced probability of a visual signal.^[41]^ Knowles found that absorption of red light in chicken photoreceptors increased as serum concentrations of chloride ion increased.^[42]^ Venkatesh et al further suggested that ion level changes during the menstrual cycle could affect visual sensitivity.^[43]^ Others have investigated the effects of blood flow on the suppression of long-wavelength cone responses to an intense 640 nm (i.e., red) stimulus and found that flicker suppression was related to heart rate but inversely related to blood pressure.^[44]^ It is possible then to attribute a portion of the present difference in B/L ratios for the red stimulus to either cyclical temperature, ionic, or blood flow changes that occur in cycling women but not contraceptive users.

While the present effects of hormonal contraception and menstrual cycle phase for the red stimulus are interesting, the lack of effects for the blue stimulus are somewhat surprising. The blue stimulus was the most saturated of the stimuli, and B/L ratios (which depend heavily on saturation) are approximations of the H–K effect. In addition, the balance of previous hormonal effects was for short-wavelength stimuli.^[14,15][32]^ When unplanned simple correlations between B/L ratios (simple approximations of the H–K effect) and estradiol (E2) or progesterone (P) levels were examined at each wavelength, there were no significant bivariate relationships for all subjects combined. However, correlations between B/L ratios and E2 × *P* were negative for all stimuli for cycling women (range –0.368 to –0.026; *P* > 0.05). This compares with previous results that implicate high E2 and P levels (during the luteal phase) in decreased sensitivity to short-wavelength stimuli during visual field testing.^[14,15]^However, correlations were positive (range, 0.314 to 0.681) for the present OC users and significantly so for the 450 nm stimulus (*P* = 0.02). It is likely that the visual mechanisms transforming short-wavelength stimuli react differently to the synergism (or antagonism) of endogenous estradiol and progesterone than to that of synthetic estradiol and progestin.

### Hormone Implications

This present finding for the blue stimulus is adjacent to a previous report that about one-third of healthy women described a short-wavelength (440 nm) patch as “white” more often than “blue” or “lavender.”^[32]^ In addition, most subjects using estrogen modulator therapy (i.e., breast cancer adjuvant therapies such as Tamoxifen) also referred to the patch as “white” more often than peri- or post-menopausal middle-aged control subjects not using any hormonal therapies. The experimental dynamics of that investigation were vastly different from the current study, using a threshold level “blue” stimulus against an adaptive yellow background, whereas the luminosity in supra-threshold test stimuli was measured in relatively young, pre-menopausal participants in the present study. The balance, however, of similar studies of menopause,^[45,46]^ OC use,^[33,34]^ or estrogen modulator therapies^[14,15][32]^have revealed consistent decreases in sensitivity to short-wavelength stimuli. While variations in shortwave-sensitive mechanisms in menopausal women could be due to the yellowing (and “blue-blocking”) of the aging crystalline lens, the differences in younger subjects are most likely due to hormonal (specifically, estrogen) effects. For example, Eisner and Samples observed that both peri- and fully menopausal women with diets rich in flax or soy (i.e., phytoestrogens) had improved short wavelength automated perimetry (SWAP) performance.^[45]^ While much of the evidence is not current, hormone disruption during OC use has long been implicated in vision (specifically short-wavelength or blue) deficits.^[33,34][47]^ Fine and McCord^[33]^ and Marre et al^[34]^ observed acquired tritanomalous (i.e., blue-yellow axis) defects with certain estrogen-based OC. Lakowski and Morton further revealed blue-yellow and blue-green deficits as well as red-green changes in OC users.^[47]^ In sum, the present findings are related to and add to the body of evidence that between subject (i.e., by contraceptive use) and within subject (i.e., hormone level) differences affect the brightness–luminance relationship for long and short wavelength stimuli.

While the modelled differences between cycling women and OC users represent small (threshold-level) differences in modelled brightness, these results build on previous results that found clinically relevant threshold level differences in chromatic stimuli across the menstrual cycle^[14]^ and for modulated estradiol.^[32]^ The question remains: Why are the effects greater for blue and red stimuli? In a gross sense, it may be simply anatomical. That is, as luminance processing results from the addition of medium- (i.e., green) and long- (i.e., red) wavelength sensitive mechanisms,^[20]^ it is simplistic—but reasonable—to think of achromatic signalling to be minimum and chromatic/achromatic ratios to be maximum at the saturated ends of the visible spectrum (i.e., blues and reds). This can be appreciated in the approximate representation of the H–K effect shown in Figure 1B, where the difference in perceived brightness between high and low saturation conditions is more dependent on chromatic differences for the blue and red stimuli. A potential explanation for cyclical changes in the H–K effect—and particularly those for blue and red stimuli—may then be based in the previous claim that fewer neuro-retinal cells serve chromatic pathways than luminance pathways.^[48,49]^Chronic estradiol administration in post-menopausal women is associated with decreased microvasculature resistance,^[50]^ but it is opposed by progesterone which is secondarily associated with vasoconstriction of retinal vessels.^[51]^ As estrogen receptors are found in most retinal layers as well as in retinal ganglion cells,^[28]^ variations in estrogen could differentially penalize the chromatic signal and result in a decreased chromatic contribution to brightness.

Regarding limitations, inferences from the present results are most limited by the low participation rate. Due to the expectations on participants, this was difficult to avoid. Second, and most importantly, E2 peaks predicted for the peri-ovulatory phase in cycling women were missed [Figure 2A]. This result has been seen previously^[52]^ and may be avoided in future studies with inexpensive at-home urine tests that help provide an accurate method of verifying peri-ovulation timing and estrogen surges.^[53]^ Lastly, salivary hormone levels may well represent circulating bioavailable hormones,^[54]^ but commercially available salivary hormone assays underestimate hormones in OC users.^[36]^ However, while this may significantly affect inferences about models including hormones, it would not affect luminance and saturation models which both clearly differ by contraceptive use.

In summary, these results provide clear support for separating cycling women from OC users in studies of brightness, particularly when luminance and saturation are predictors. Second, they add sex hormones to current brightness models for cycling women. Lastly, the interaction effect of OC use and MC phase on B/L ratios for the red stimulus adds to a rich history of long-wavelength mechanisms. All these results deserve additional consideration.

##  Financial Support and Sponsorship

None.

##  Conflicts of Interest

None.
